# Rapid Assessment of Non-Volant Mammals in Selected Areas of Peninsular Malaysia

**DOI:** 10.21315/tlsr2025.36.1.8

**Published:** 2025-03-30

**Authors:** Hannah Syakirah Ab Hamid, Nur Dayana Zulkifli, Mazrul Aswady Mamat, Amirrudin Ahmad, Nobuyuki Yamaguchi, Nurulhuda Zakaria, Hafizan Juahir, Muhamad Safiih Lola, Mohd Tajuddin Abdullah

**Affiliations:** 1 Faculty of Science and Marine Environment, Universiti Malaysia Terengganu, 21030 Kuala Nerus, Terengganu, Malaysia; 2 Institute of Tropical Biodiversity and Sustainable Development, Universiti Malaysia Terengganu, 21030 Kuala Nerus, Terengganu, Malaysia; 3 East Coast Science and Environmental Research Institute, Universiti Sultan Zainal Abidin, Gong Badak Campus, 21300 Kuala Nerus, Terengganu, Malaysia; 4 Faculty of Bioresources and Food Industry, Universiti Sultan Zainal Abidin, Besut Campus, 22200 Besut, Terengganu; 5 Faculty of Science Computer and Mathematic, Universiti Malaysia Terengganu, 21030 Kuala Nerus, Terengganu, Malaysia; 6 Faculty of Fisheries and Food Sciences, Universiti Malaysia Terengganu, 21030 Kuala Nerus, Terengganu, Malaysia; 7 Academy of Sciences Malaysia, Level 20, West Wing, MATRADE Tower, Jalan Sultan Haji Ahmad Shah, off Jalan Tuanku Abdul Halim, 50480 Kuala Lumpur, Malaysia

**Keywords:** Diversity, Island, Lake, Urban, Wetland, Habitat, Non-volant Mammals, Diversiti, Pulau, Tasik, Bandar, Tanah Bencah, Habitat, Mamalia Bukan Terbang

## Abstract

Non-volant mammals in Peninsular Malaysia face numerous threats, primarily driven by habitat loss, fragmentation and illegal hunting. These threats highlight the importance of conducting wildlife surveys in the available forested areas to enhance the current strategies for conservation and management, particularly for a threatened taxon like non-volant mammals. This study aimed to document and update information of non-volant mammals from four areas: Tasik Bera (Pahang state); Tasik Kenyir, Pulau Redang and Universiti Malaysia Terengganu (UMT) Campus (Terengganu state). Cage traps and Visual Encounter Survey methods were utilised to record non-volant mammals from August 2022 until March 2023. This study successfully documented 123 individuals from 27 non-volant mammal species, representing 11 families from 6 orders. Tasik Bera demonstrated the highest species count at 18, followed by UMT Campus with 6, while Tasik Kenyir and Pulau Redang each recorded 4 species. The species diversity was the highest at Tasik Bera (*H′* = 2.65) and the lowest at Pulau Redang (*H′* = 1.01). *Macaca fascicularis*, *Tupaia glis* and *Paradoxurus hermaphroditus* were recorded from three of four sites. This study has added new geographically recorded species for Tasik Bera (11 species) and UMT Campus (4 species). This study has advanced our knowledge of the diversity and distribution of non-volant mammals, enhancing our understanding in this field. This understanding is crucial for implementing efficient conservation and management strategies, aiding in the development of targeted conservation strategies to protect these species and their habitats.

## Abstract (fr)

**Abstrak:**

Mamalia bukan terbang di Semenanjung Malaysia menghadapi pelbagai ancaman, terutamanya disebabkan oleh kehilangan habitat, pemecahan habitat dan pemburuan haram. Ancaman ini menekankan kepentingan menjalankan tinjauan hidupan liar di kawasan hutan yang masih tersedia untuk meningkatkan strategi pemuliharaan dan pengurusan semasa, khususnya bagi takson yang terancam seperti mamalia bukan terbang. Kajian ini bertujuan untuk mendokumentasikan dan mengemas kini maklumat mengenai mamalia bukan terbang dari empat kawasan: Tasik Bera (negeri Pahang); Tasik Kenyir, Pulau Redang, dan Kampus Universiti Malaysia Terengganu (UMT) (negeri Terengganu). Kaedah perangkap sangkar dan Tinjauan Penemuan Visual digunakan untuk merekodkan mamalia bukan terbang dari Ogos 2022 hingga Mac 2023. Kajian ini berjaya mendokumentasikan 123 individu daripada 27 spesies mamalia bukan terbang, mewakili 11 famili daripada 6 order. Tasik Bera mencatat jumlah spesies tertinggi dengan 18 spesies, diikuti oleh Kampus UMT dengan 6 spesies, manakala Tasik Kenyir dan Pulau Redang masing-masing merekodkan 4 spesies. Kepelbagaian spesies adalah tertinggi di Tasik Bera (*H′* = 2.65) dan paling rendah di Pulau Redang (*H′* = 1.01). *Macaca fascicularis*, *Tupaia glis* dan *Paradoxurus hermaphroditus* direkodkan di tiga daripada empat tapak kajian. Kajian ini telah menambah rekod spesies baharu dari segi geografi untuk Tasik Bera (11 spesies) dan Kampus UMT (4 spesies). Kajian ini telah memperluas pengetahuan mengenai kepelbagaian dan taburan mamalia bukan terbang, seterusnya meningkatkan pemahaman dalam bidang ini. Pemahaman ini amat penting bagi pelaksanaan strategi pemuliharaan dan pengurusan yang berkesan, serta membantu dalam pembangunan strategi pemuliharaan yang disasarkan untuk melindungi spesies ini dan habitatnya.

**Highlights**

- A total of 123 individuals, representing 27 non-volant mammal species from 11 families and six orders were documented.
- Numerous mammal species including primates, elephants and carnivores face critical conservation challenges, with WCA 2010 protection and IUCN Red List statuses from Critically Endangered to Vulnerable.
- 15 species were documented as new geographically records for the area and add to the existing knowledge on mammalian distribution in Malaysia.

## INTRODUCTION

Malaysia is widely renowned for its high degree of biodiversity, as it is one of the world’s 17 megadiverse countries, having 440 species of mammals (Department of Wildlife and National Park 2016). Among these, approximately 15% (66 species) are endemic to Malaysia (Payne *et al*. 1998; Francis & Barrett 2008). Malaysia has a wide array of natural wonders and ecological treasures, spanning the Peninsular and the Borneo region. Its exceptional biodiversity encompasses lush rainforests, majestic waterfalls, winding rivers, serene lakes and reservoirs, expansive oceans and seas, and diverse flora and fauna, creating a harmonious balance of biotic and abiotic elements (Abdullah *et al*. 2017).

In 2021, Peninsular Malaysia’s forested area amounted to 5.73 million hectares, including a permanently conserved forest of 4.85 million hectares (Forestry Department of Peninsular Malaysia 2022). However, much unprotected land has been converted for human settlements, recreational activities, tourism, agriculture and industrial parks. Despite Malaysia’s ecological richness, non-volant mammals face threats, particularly due to rapid expansion of industrial agriculture (Razali *et al*. 2018), tourism (Abdullah *et al*. 2011), extensive fragmentation (Lane *et al*. 2006), human disturbances and anthropogenic activities (Struebig *et al*. 2008) and habitat loss (Kingston *et al*. 2003). Consequently, the number of non-volant mammal species has declined over the years as human activities disturb the environment (Crooks *et al*. 2017). Conservation efforts for non-volant mammals in Peninsular Malaysia are crucial due to the increasing anthropogenic pressures and habitat degradation (Baharudin *et al*. 2023). Assessing the conservation status of these species is essential for effective management and conservation planning, with such assessments aid in prioritising conservation efforts and formulating conservation strategies to safeguard these vulnerable species. Understanding non-volant mammals’ ecological requirements and interactions is crucial for their conservation and management (Lacher *et al*. 2019).

There have been many studies on the diversity of non-volant mammals that were conducted in various types of habitats in Peninsular Malaysia such as on islands (Abdullah *et al*. 2019; Baqi *et al*. 2021; Rahim *et al*. 2016; Tamblyn *et al*. 2005; Zahidin *et al*. 2022), lakes (Afiq Ramlee *et al*. 2020; Madinah *et al*. 2011; Khalib *et al*. 2018; Syakirah *et al*. 2000; William-Dee *et al*. 2019), forests (Zakaria *et al*. 2001; Saiful *et al*. 2001; Ramli & Hashim 2009; Shahfiz *et al*. 2011; Jayaraj *et al*. 2012; Ahmad Juffiry *et al*. 2015; Baharudin *et al*. 2023) and mangroves (Anuar 2007; Mohd Nasir 2008; Samsudin 2007). These preceding studies have indeed yielded valuable insights and significant findings. However, they may now be outdated, leaving a knowledge gap in current knowledge. In the 2017 IUCN Red List assessment for Peninsular Malaysia, 219 out of 223 listed mammal species were evaluated. Results show four species was assessed as Critically Endangered, 13 species as Endangered, 28 species as Vulnerable and 26 species as Near Threatened. Additionally, one species was classified as Extinct, four species as Critically Endangered, 12 species as Endangered, 14 species as Vulnerable and 34 species as Near Threatened (PERHILITAN 2017).

The objectives of this study were:

1. To determine the diversity of non-volant mammals in four different sites.
2. To compile and update the taxonomic checklist of non-volant mammals for each study site.
3. To assess the status of non-volant mammal communities at the sites.

This study offers a chance to explore non-volant mammal distribution and ecological roles in various habitats, enhancing our understanding of mammal diversity in Malaysia. The data collected can inform biodiversity conservation and sustainable management practices, aiding policymakers and planners. This study will help develop strategies to mitigate human activities involves a combination of conservation actions, policy interventions, community engagement and scientific research to safeguard mammal species and their habitats, ensuring the coexistence of mammals and their habitats.

## MATERIALS AND METHODS

### Study Sites

**Figure 1:**
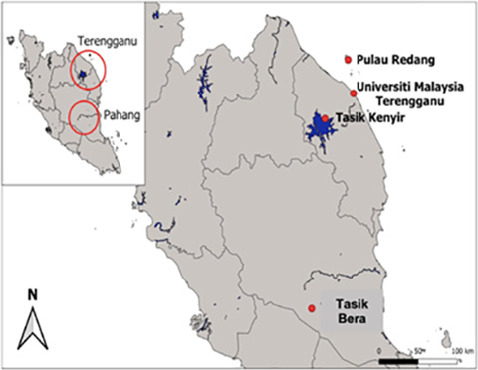
Map of the study sites. The red dots represent the location of present study sites. (*Source*: Created using GIS software)

**Figure 2:**
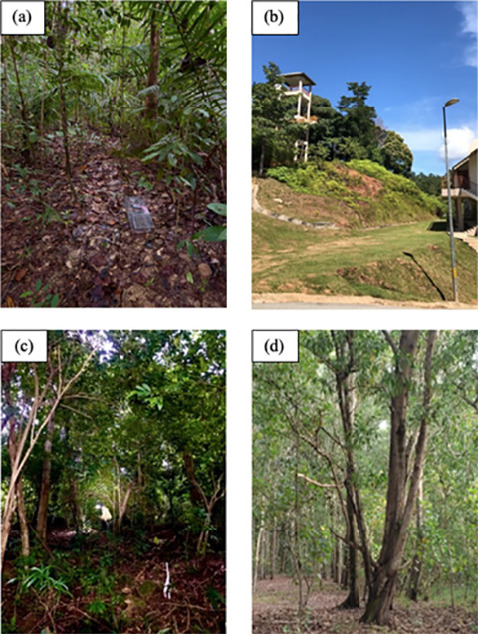
Location of trapping sites with (a) Tasik Bera, (b) Tasik Kenyir, (c) Pulau Redang and (d) UMT Campus.

Tasik Bera Ramsar Site, Tasik Kenyir, Pulau Redang and Universiti Malaysia Terengganu (UMT) campus were selected as sampling sites between August 2022 and March 2023 (Table 1; Fig. 1; Fig. 2). Tasik Bera, situated in Pahang state is a significant freshwater ecosystem, the largest natural lake in Peninsular Malaysia (Gharibreza *et al*. 2013). Declared Malaysia’s first Ramsar site in 1994, it spans 35 km by 20 km and covers 7,000 ha of diverse habitats (Biun & Mohd Buang 2014). Tasik Bera is a complex interlocking ecosystem made up of open waters, reedbeds, lakes and rivers surrounded by a patchwork of dry lowland dipterocarp forest (Biun & Mohd Buang 2014). These habitats support a complex web of interactions among plants, animals and microorganisms, contributing to the ecosystem’s overall ecological functioning and resilience. Despite its ecological importance, Tasik Bera faces several conservation challenges. The expansion of human activities including agriculture, aquaculture and tourism has led to habitat degradation and loss (Henson 1994; Sharip & Zakaria 2008; Gharibreza *et al*. 2013). This research focuses on the lowland primary forest habitat near the Management Office of the Tasik Bera Ramsar Site.

Tasik Kenyir is situated in Hulu Terengganu district near the Kelantan border between 30° 31 N and 102° 3 E in the low-lying, undulating plain between the Main Range in the West and hill ranges to the East (Norfaizal *et al*. 2015). Tasik Kenyir is one of the largest artificial lakes in Southeast Asia, spanning 209,199 ha, surrounded by a total of 4,975 ha of tropical forest, with 340 islands (Mohammad Noor *et al*. 2019). The islands on Tasik Kenyir are the tips of hilltops which were not submerged during the flooding process. The study took place at the UMT Research Station within the Tasik Kenyir area.

Pulau Redang, one of the largest islands in Peninsular Malaysia lies approximately 45 km east of Kuala Terengganu. It was designated an interim Fisheries Prohibited Area (FPA) in early 1983, extending 8 km offshore, protecting marine life from unauthorised harvesting. The Pulau Redang Marine Park Centre, established in 1990, safeguards the island’s diverse marine and terrestrial ecosystems. The research was carried out near the Delima Redang Resort.

At a coastal plain in UMT campus in Mengabang Telipot, Kuala Nerus, the study was conducted along a 2 km boardwalk called Jalan Baywalk. The plain contains areas of muddy swales, which were previously lagoons but have now transformed into wetlands. A thick coastal mangrove forest previously covered this wetland area, but some have now been converted into urban areas (Badli Sham *et al*. 2019).

### Sampling Method

At Tasik Bera, 16 cage traps with dimensions of 42 cm × 16 cm × 16 cm were utilised, while at Tasik Kenyir, Pulau Redang and UMT Campus, 10 cage traps were set up during each sampling session. The types of bait varied, with oil palm fruit being used as bait at Tasek Bera, while banana was the bait of choice at Tasik Kenyir, Pulau Redang and the UMT Campus. The choice of bait for non-volant mammals varied between study sites due to the unique ecosystems and specific vegetation types harboured by each location, which influenced the availability of natural food sources for these mammals. For example, Tasik Bera had a higher abundance of oil palm fruit due to nearby plantations, making it a readily available and attractive bait option. Conversely, locations such as Tasik Kenyir, Pulau Redang and the UMT Campus had different vegetation types where bananas were more abundant and easily accessible. Moreover, non-volant mammals had diverse dietary preferences, and the choice of bait was often tailored to attract the target species efficiently. Some species showed a strong preference for certain fruits or food items over others. Therefore, this study selected baits that were most likely to attract the desired species based on their known dietary habits and preferences. In this case, oil palm fruit and bananas were likely chosen because they were known to be consumed by a wide range of non-volant mammals in the respective study areas.

The cage traps were randomly placed on the ground and at a height of 5 m on tree branches along the existing trails, with 20 m between each cage. The traps were checked twice daily at 0800 h and 1700 h (Rahim *et al*. 2016), and the baits were renewed daily after checking. The cage traps were repositioned within the sampling sites every two days to expand the coverage of the sampling area and enhance the likelihood of capturing a greater number of individuals. All the small mammals caught were carefully removed from the traps and placed temporarily in a cloth bag. Captured animals were immobilised using mild chloroform, and measurements such as weight, four morphological measurements (HB = Head body length, TL = Tail length, HF = Hind foot length and E = Ear length) and sex were recorded (Payne *et al*. 1985). Species identification was done by referring to Medway (1983), Francis (2008) and Francis and Barrett (2008). All captured animals were promptly released back into their natural habitats on the same day following capture.

Throughout the study, Visual Encounter Surveys (VES) were meticulously conducted through both on-foot traverses along designated transects and mobile surveys via vehicle, specifically in Tasik Bera. A comprehensive total distance of 294 km was covered within Tasik Bera, complemented by 1.68 km in Tasik Kenyir, 23.09 km around Pulau Redang and 52.9 km across the UMT Campus. Observations of animals were documented utilising binoculars and spotlights. Each sighting was photographed, with accompanying records detailing the number of individuals and their species identification carried out with reference to Medway (1983), Francis (2008) and Francis and Barrett (2008).

## DATA ANALYSIS

The species diversity indices, including Shannon Diversity Index (*H′*), Evenness Index (*E*) and Simpson Dominance Index (*D*) were calculated using the Paleontological Statistics (PAST) program (Hammer *et al*. 2001). Species accumulation curves (SACs) were computed using EcoSim (Gotelli & Entsminger 2015) to deduce the completeness of the inventory for non-volant mammal assemblages in the four study sites. Venn diagrams were created to visually show unique or common species among different study sites and demonstrate the similarity and overlap of species composition. Trapping effort and capture rate were measured at each site to evaluate trapping methods’ effectiveness and the target species’ abundance.

## RESULTS

**Figure 3:**
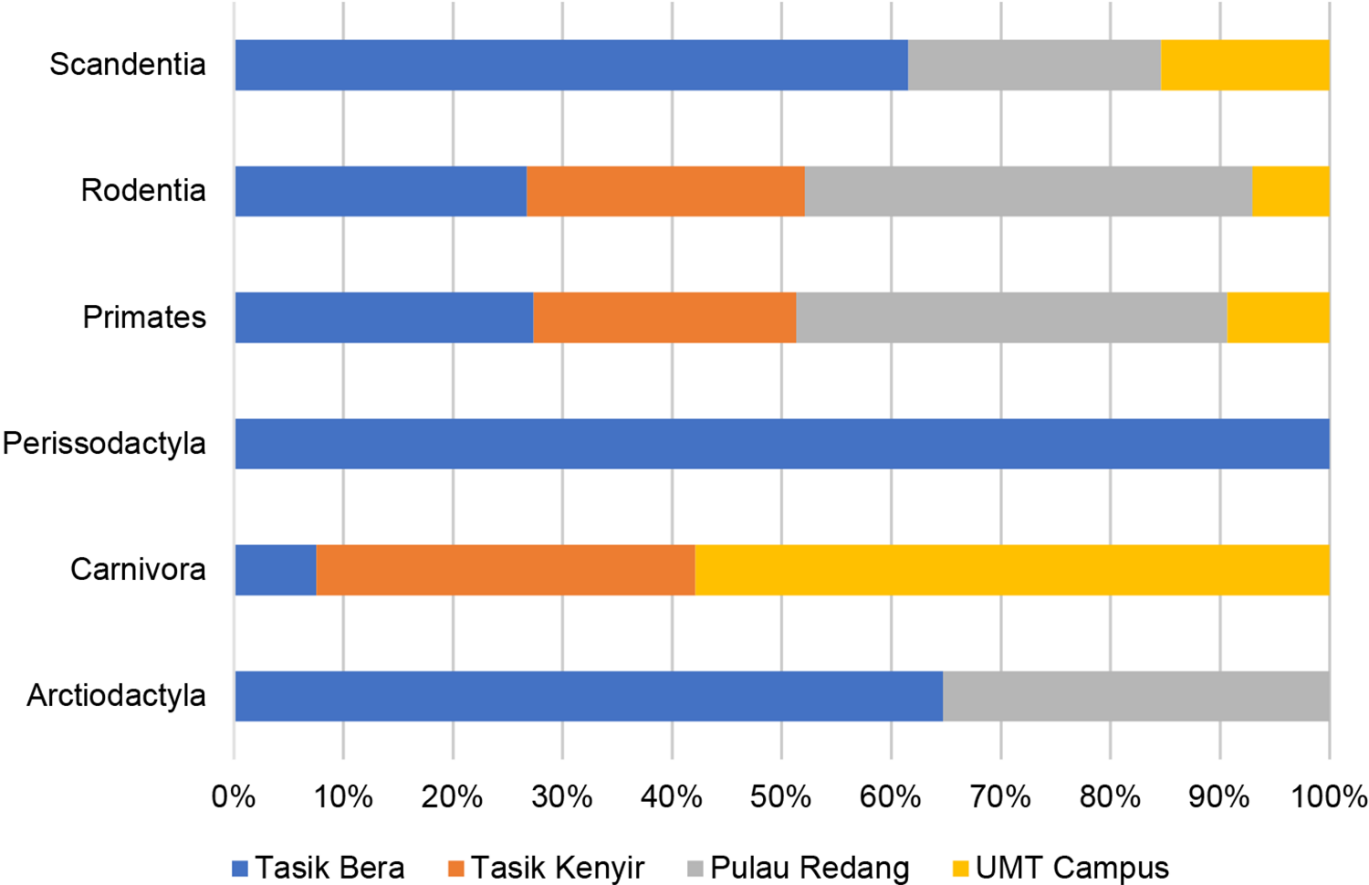
The distribution of non-volant mammals at Tasik Bera, Tasik Kenyir, Pulau Redang and UMT Campus, categorised by order.

**Figure 4:**
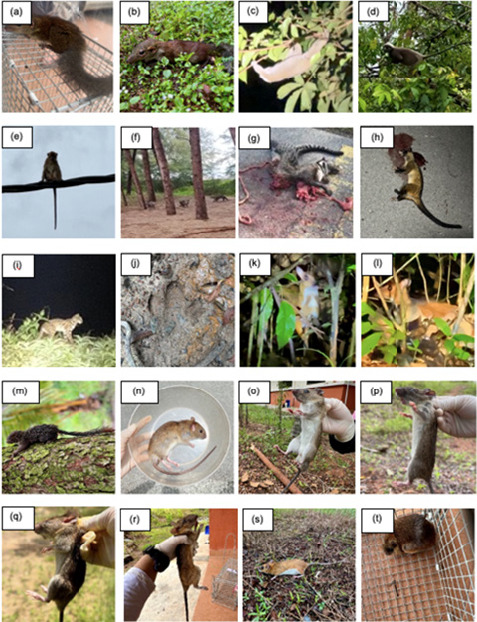
Non-volant mammals species with (a) *Tupaia minor*, (b) *Tupaia glis*, (c) *Nycticebus coucang*, (d) *Trachypithecus obscurus*, (e) *Macaca fascicularis*, (f) *Lutrogale perspicillata*, (g) *Viverra tangalunga* roadkill, (h) *Paradoxurus hermaphroditus* roadkill, (i) *Prionailurus bengalensis*, (j) *Tapirus indicus* footprint, (k) *Tragulus kanchil*, (l) *Tragulus napu*, (m) *Callosciurus notatus*, (n) *Rattus rattus*, (o) *Rattus tiomanicus*, (p) *Rattus argentiventer*, (q) *Rattus exulans*, (r) *Leopoldamys sabanus*, (s) *Maxomys rajah* and (t) *Maxomys whiteheadi*.

During the designated sampling periods, a rapid assessment across the four study sites successfully documented six orders (Fig. 3). At Tasik Bera, Primates exhibited the highest percentage, accounting for 41% of the observed mammals, followed by Rodentia (19%), Scandentia (16%), Artiodactyla (11%), Carnivora (10%) and Perissodactyla (3%). Meanwhile, Pulau Redang and UMT Campus observed four orders, with Primates (59%) and Carnivora (77%) being the predominant orders in each respective area. In contrast, Tasik Kenyir recorded only three orders, with Carnivora (46%) being the most prominent order during the sampling period, followed by Primates (36%) and Rodentia (18%). This study successfully documented 123 individuals from 27 non-volant mammal species, representing 11 families from six orders (Fig. 4; Table 2). Notable among these families, the Viverridae family exhibited the highest number of species, with four species spanning four genera, *Arctictis*, *Paguma*, *Paradoxurus* and *Viverra*. Subsequently, the Cercopithecidae family encompasses three species, while the Muridae and Felidae families each encompass three species. Additionally, the Sciuridae and Tupaiidae families each comprise two species, followed by the Elephantidae, Lorisidae, Mustelidae, Tapiridae and Tragulidae families, each with a single species representation.

The species most often recorded was *Macaca fascicularis* with 28 independent observations. These were predominantly observed in the Tasik Bera, constituting 54% (15 observations) of the overall count. Additionally, a smaller group was observed on Pulau Redang (10 observations) and the UMT Campus (3 observations). A more notably observed species, *Tupaia glis* was documented with a cumulative count of 12 observations, primarily captured through cage traps. This distribution was skewed towards Tasik Bera, where 11 observations were recorded, while one individual was recorded on Pulau Redang. Six species were recorded as single individuals caught or observed including *Tupaia minor*, *Paguma larvata*, *Arctictis binturong*, *Rattus rattus*, *R. tiomanicus* and *Maxomys whiteheadi*. According to the IUCN Red List of Threatened Species (2023), several species recorded in Tasik Bera exhibit varying conservation statuses (Table 2). In terms of global conservation status, *Presbytis femoralis* is classified as Critically Endangered (CR), while *Nycticebus coucang*, *Macaca nemestrina*, *M. fascicularis*, *Elephas maximus*, *Trachypithecus obscurus* and *Tapirus indicus* are classified as Endangered (EN). Additionally, other species such as *Panthera pardus*, *Maxomys rajah*, *M. whiteheadi*, *Lutrogale perspicillata* and *Arctictis binturong* are listed as Vulnerable (VU). In Peninsular Malaysia, as outlined by the Red List of Mammals for Peninsular Malaysia (PERHILITAN 2017), various species including *Panthera pardus* and *Tapirus indicus* are classified as Endangered (EN), while *Elephas maximus* is classified as Vulnerable (VU) and several others are categorised as Near Threatened (NT) or Least Concern (LC). Under the Wildlife Conservation Act (WCA 2010), certain species are designated as Protected (P) or Totally Protected (TP), with others considered Not Protected (NP).

**Figure 5:**
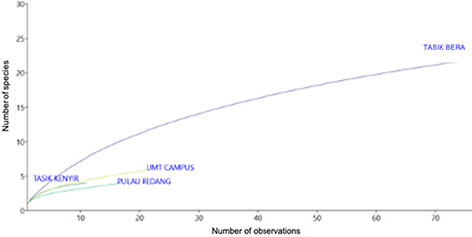
Species Accumulation Curve (SAC) for four study sites.

Tasik Bera demonstrated the highest number of species and individual, with 73 individuals from 18 species. This was followed by the UMT Campus area, which documented 22 individuals from six species. In contrast, Tasik Kenyir recorded 11 individuals representing four species, while Pulau Redang accounted for 17 individuals from four species. The Species Accumulation Curve (SAC) visually represents the cumulative number of species discovered during sampling activities within different sites. SACs in Fig. 5 shows that all four study sites have yet to reach the asymptote point, with Tasik Bera exhibits an increasing trend, indicating a rise in discovered species. However, it has not yet reached an asymptote. This suggests that there may still be additional species yet to be uncovered, emphasising the need for further sampling efforts. The SAC for Tasik Kenyir displays an almost linear curve, implying that the sampling session may have reached asymptote, indicating that most species in the area have likely been discovered. Conversely, the SACs for UMT Campus and Pulau Redang indicate that additional sampling efforts are necessary to comprehensively understand the non-volant mammal species in those areas. No species are shared among all four study sites.

Tasik Bera harbours the highest number of unique species (16 species), followed by Tasik Kenyir (two species), UMT Campus (three species) and no unique species was identified in Pulau Redang. Among the species observed, *Tupaia glis* and *Macaca fascicularis* were recorded in three of the four sites, Tasik Bera, Pulau Redang and UMT Campus. *Paradoxurus hermaphroditus* was also found in three of the four sites, specifically Tasik Bera, Tasik Kenyir and UMT Campus. Additionally, Tasik Bera recorded the highest trapping effort of 160 trap days (Table 3). This site’s capture rate of 0.46 individuals per trapping effort of all trapped species combined indicates relatively effective trapping success as compared to the other sampling sites. Moreover, it displayed the highest level of species diversity with a Shannon Diversity Index, *H′* = 2.65, and a Simpson Dominance index, *D* of 0.91, implying balanced species distribution. Tasik Kenyir, Pulau Redang and UMT Campus displayed relatively lower capture rates. These sites showed lower diversity indices in comparison to Tasik Bera, with Pulau Redang recording the lowest values (*H′* = 1.01), indicating reduced diversity and potentially a skewed species composition (*D* = 0.6).

### Comparison with Previous Studies

The findings of this study were compared with those of prior research investigating non-volant mammals across selected locations, specifically Tasik Bera, Tasik Kenyir, Pulau Redang and UMT Campus (Table 4). There are notable variations in non-volant small mammal species diversity across the four study sites. In this study, Tasik Bera documented a lower count in comparison to the work of Syakirah *et al*. (2000), who reported 33 species across 13 families. In the context of other areas such as Tasik Kenyir and Pulau Redang, this study recorded the fewest species compared to previous studies. In Pulau Redang, the earliest documentation by Robinson and Kloss (1911) reported six species from five families. However, recent studies on the UMT Campus have revealed a higher number of species compared to earlier investigations by Samsudin (2007), Anuar (2007) and Mohd Nasir (2008), recording six species from five families. In the case of Tasik Kenyir, Afiq Ramlee *et al*. (2020) documented the highest number of species count to date, recording a total of 91 species from 13 orders.

**Figure 6:**
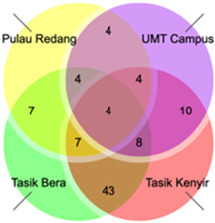
Venn diagram showing numbers of shared species between Tasik Bera, Tasik Kenyir, Pulau Redang and UMT Campus. The number of species is shown in each of the subsets.

By combining all available records, Tasik Kenyir showcased the highest number of species, with 87 species belonging to 62 genera and 24 families. In contrast, Pulau Redang and UMT Campus showed relatively lower number of species, with only 9 and 10 species observed, respectively. Tasik Bera demonstrated intermediate number of species, with 47 species belonging to 34 genera and 16 families. Among the species observed, several exhibited restricted distributions, with certain species found exclusively at specific study sites. For instance, *Bos gaurus* was present only at Tasik Kenyir, while *Capricornis sumatraensis* and *Rusa unicolor* were exclusively found at Tasik Kenyir. Fig. 6, depicted in a Venn diagram illustrates the shared species across the four study sites. Notably, Tasik Bera and Tasik Kenyir exhibited the highest level of species overlap, with 43 shared species. Key species such as *Macaca fascicularis*, *Rattus tiomanicus*, *Callosciurus notatus* and *Tupaia glis* were consistently observed across all sampling sites. Additionally, *Crocidura fuliginosa* was exclusively present on an isolated island within Pulau Redang, while *Nycticebus coucang*, *Lenothrix canus* and *Ptilocercus lowii* were solely documented within Tasik Bera.

## DISCUSSION

The results of this study provide valuable updates into the non-volant mammal community in the selected sites during the sampling period. Tasik Bera documented the highest number of species (22 species), followed by the UMT Campus (6 species) and Tasik Kenyir and Tasik Bera (4 species each). Tasik Bera and UMT Campus have contributed new geographically recorded species. Specifically, Tasik Bera has added 11 species namely *Presbytis femoralis*, *Trachypithecus obscurus*, *Macaca nemestrina*, *M. fascicularis*, *Paradoxurus hermaphroditus*, *Panthera pardus*, *Elephas maximus*, *Tapirus indicus*, *Tragulus kanchil*, *T. napu* and *Rattus argentiventer*. By comparison, three species, *Leopoldamys sabanus*, *Maxomys rajah* and *M. whiteheadi* are shared between Tasik Bera and previous studies. UMT Campus has also documented four new geographically recorded species namely *M. fascicularis*, *Lutrogale perspicillata*, *Paguma larvata* and *Tupaia glis*. Additionally, UMT Campus shares one species with previous studies, *P. hermaphroditus*. Conversely, neither unique nor shared species are observed for Tasik Kenyir and Pulau Redang compared to previous research.

The observed disparities in non-volant mammal species diversity across the study sites, including data from previous studies can be attributed to variations in habitat types, environmental conditions and levels of anthropogenic disturbances. Tasik Kenyir, characterised by extensive forest cover and diverse microhabitats, supported the highest species richness, reflecting its importance as a biodiversity hotspot. In contrast, Pulau Redang and UMT Campus, subjected to greater human impact and habitat fragmentation, exhibited reduced species diversity. The presence of species such as *Bos gaurus*, *Capricornis sumatraensis*, *Rusa unicolor* and *Muntiacus muntjak* exclusively at Tasik Kenyir highlights the importance of this site for the conservation of rare and endemic species. Overall, these findings underscore the need for targeted conservation efforts to preserve the unique biodiversity of each study site and mitigate threats to non-volant mammal populations.

Despite the presence of oil palm plantations surrounding the Tasik Bera, substantial portions of undisturbed forest habitat have been preserved. These undisturbed forest areas, in conjunction with the plantation areas adjacent to the forest, collectively contribute to the high species diversity observed in Tasik Bera. In addition to the primary habitats of freshwater and peat swamp forests, there are lowland forests that encompass the lake area that are protected and can also contribute to the maintenance of a diverse range of species around it (William-Dee *et al*. 2019). The high diversity of non-volant mammals in Tasik Bera may also result from the existence of diverse microhabitats such as fallen tree logs, burrows and tree hollows, which serve as potential shelters and nesting sites (Mohammad Noor *et al*. 2019). Additionally, the diversity of habitats plays a crucial role in shaping species compositions within an area, alongside factors like the availability and distribution of food resources (Zakaria & Nordin 1998).

Furthermore, the notable disparity in number of species observed at Tasik Bera in comparison to the other three study sites may be attributed to the intensified sampling efforts deployed in the area. With an extensive span of 10 trapping days and the utilisation of a substantial number of cage traps amounting to 16, the sampling session at Tasik Bera undoubtedly contributed to the heightened species documentation. At Tasik Bera, oil palm fruit was used as bait in the cage traps, resulting in excellent capture rates of non-volant mammals, revealing wide diversity with 26 individuals from nine species: *Tupaia minor*, *T. glis*, *Callosciurus notatus*, *Rattus tiomanicus*, *R. argentiventer*, *R. exulans*, *Leopoldamys sabanus*, *Maxomys rajah* and *M. whiteheadi*. This is also likely due to the presence of oil palm plantations surrounding the Tasik Bera, which attracts animals with its abundant fruit as food sources (Syakirah *et al*. 2000). The proximity of the oil palm plantations to the Tasik Bera likely influences the movement and foraging behaviour of the animals, could lead to a higher capture rate in the cage traps. Conversely, the remaining three study sites employed a methodology consisting of 10 cage traps baited with banana slices, and the sampling duration was limited to only four days, except for the UMT Campus, where the samplings were conducted on three separate occasions. However, Visual Encounter Surveys were uniformly conducted across all four study sites. Increased sampling effort is necessary as there is a positive relationship between species richness estimates and sampling effort, where greater sampling efforts typically result in higher richness, also known as the species-sampling effort relationship (SSER) (Azovsky 2011). Although some part of Tasik Kenyir was regenerated forest as the forest was logged previously (Mohammad Noor *et al*. 2019), the species diversity is low due to weather and limited access to the forest. In Pulau Redang, vegetation clearing and tourism development (Tamblyn *et al*. 2005) consequently affect the diversity of mammals. In addition, its habitat is far from the mainland and small patches of forest on the island may limit the food sources for the animals (Hadley *et al*. 2014). UMT Campus used to have a thick coastal mangrove forest, but most of it has now been converted into urban areas (Badli Sham *et al*. 2019). Human activities and disruptions may impact the non-volant mammal community at the UMT Campus.

Most species documented in Tasik Kenyir are common non-volant mammals found in the lowland dipterocarp forests of Peninsular Malaysia, as highlighted by Ruppert *et al*. (2015). These species play crucial roles as seed dispersers, particularly evident among species from the families of Muridae, Sciuridae and Tupaiidae. Studies have indicated that the faeces of these mammals often contain fig seeds (*Ficus* sp.), underscoring their importance as dispersers of seeds (Wells *et al*. 2009; Wells & Bagchi 2005) in the lowland rainforests of Tasik Kenyir. *Maxomys rajah* and *M. whiteheadii*, belonging to the family Muridae are classified as vulnerable (VU) according to the IUCN Red List of Threatened Species (IUCN 2016). Despite being common in Southeast Asia, habitat destruction in certain regions poses a risk to their populations (Francis 2008). The enigmatic giant squirrel, *Ratufa bicolor*, from family Sciuridae is categorised as Near Threatened (NT) by the IUCN (2016) and is fully protected in Peninsular Malaysia under the Wildlife Conservation Act (2010). Conversely, *Tupaia minor* and *T. glis*, belonging to the family Tupaiidae are also protected under the Wildlife Conservation Act (2010) in Peninsular Malaysia, despite being listed as of Least Concern (LC) in the IUCN Red List of Threatened Species (IUCN 2016) and in the Red List of Mammals for Peninsular Malaysia (Department of Wildlife and National Parks 2019).

*Macaca fascicularis* and *Tupaia glis* were recorded at Tasik Bera, Pulau Redang and UMT Campus, and they were among the most observed species in the study. This observation suggests that these areas likely provide suitable habitats or resources for both species. *M. fascicularis* is known for its adaptability to various habitats, ranging from coastal areas to inland forests. They are opportunistic feeders and can thrive in diverse environments if essential resources such as food, water and suitable shelter are available (Gumert & Malaivijitnond 2012). This adaptability makes them well-suited to a wide range of habitats found in Malaysia Osman *et al*. 2022). The occurrence of *M. fascicularis* in Tasik Bera, Pulau Redang and UMT Campus suggests that these areas likely provide a combination of forested areas and open spaces, which align with the species’ habitat preferences (Holzner *et al*. 2019). Their presence across these diverse sites is supported by their ability to exploit both natural food sources and the readily available human provisions found in nearby settlements, including crops and waste (Dzulhelmi *et al*. 2019; Mun 2014). In forested areas of Tasik Bera, *M. fascicularis* display arboreal behaviour, while in urban settings of UMT Campus, they are known to adapt to ground-dwelling behaviours. At Pulau Redang, *M. fascicularis* are usually seen in troops at the edge of the forests surrounding the resorts and along trails. In the dense forests of Tasik Kenyir, where the canopy cover may be extensive and the understory vegetation dense, the habitat structure may not align with the preferences of *M. fascicularis*. This macaque may find it challenging to access the resources they need or to navigate effectively within such dense vegetation. Additionally, factors such as competition with other primate species such as *Trachypithecus obscurus* could further deter *M. fascicularis* from establishing populations in these dense forest environments.

*Tupaia glis* was also recorded at Tasik Bera, Pulau Redang and UMT Campus, with this species being the second most observed in the study. The presence of *T. glis* in these locations could indicate the availability of suitable food resources and appropriate habitat structure for their arboreal lifestyle. *T. glis* has been noted to prefer ground foraging over arboreal activities (Langham 1982). Typically, this generalist species inhabits primary dipterocarp forests, although they can endure certain levels of habitat alteration. They have also been documented in secondary forests, plantations, fruit orchards and trees near residential areas (Parr 2003). *T. glis* has been previously documented in Selangor, Kelantan, Pahang and Perak (Ruppert *et al*. 2015; Jayaraj *et al*. 2012; 2013; Tingga *et al*. 2012; Zakaria *et al*. 2001). This treeshrew exhibits a high tolerance towards habitat disturbance (Zakaria *et al*. 2001; Corlett 1992). *T. glis*, known for its monogamous and highly territorial nature, typically maintains a relatively large home range spanning several hectares. Their average total active period ranged from 4.90 to 7.00 hours, with a total daily travel distance of 270 m to 382 m (Mariana *et al*. 2010). Moreover, a male and a female treeshrews can cover distances of up approximately 3,285 m, while female treeshrews can travel distances of around 4,591 m (Mariana *et al*. 2010). This wide-ranging behaviour allows it to explore and exploit different habitats within its territory, depending on the availability of resources and suitable shelter. Additionally, the significant abundance of this ground-dwelling species may be attributed to its capability to breed at any time throughout the year, its short gestation period and the lack of restrictions to a specific breeding season (Francis 2013; Medway 1983). This animal displays a fearlessness and remains unperturbed by the presence of eco-tourists walking around beaches and trails, frequently seen near humans (Rahim *et al*. 2016). The combination of its dietary flexibility, broad home range and adaptability to varying environments makes *T. glis* a successful and widely distributed species across different habitats. The dietary preferences of this species primarily consist of fruits, seeds, leaves and insects, with a particular affinity for ants and spiders (Lim 1995; Nowak 1999). Given their dietary habits, these treeshrews play a crucial role in regulating insect populations within the study sites. By consuming insects, they contribute to controlling insect numbers, which can have a positive effect on ecosystem balance by preventing outbreaks of certain insect species. This natural pest control mechanism underscores the ecological importance of *T. glis* in maintaining the health and equilibrium of their habitats.

Tasik Bera stood out as the site with the highest presence of species from the family Muridae, with six out of the seven species recorded in this study documented there. The Muridae is the largest family of mammals, comprising over 1,300 species, and exhibits a remarkable array of adaptations for life in and around water (Pacini & Harper 2008). They require food, shelter (Witmer *et al*. 2007) or a buffer zone (Yletyinen & Norrdahl 2008), which can be provided by agricultural areas, edge forests, non-agricultural land or human dwellings, as found in Tasik Bera. *Leopoldamys sabanus* and *Maxomys rajah* are known to demonstrate scatter-hoarding behaviour (Yasuda *et al*. 2000). Food hoarding behaviour allows terrestrial rodents to optimise its foraging activities, as well as to increase the chances of survival during food insufficiency (Yasuda *et al*. 2000). This behaviour also benefits the plants by dispersion of seed (Howe *et al*. 1985; Vander Wall 1990). Thus, these species acting as seed dispersal agents are important in the extension of forest area and maintaining the quality of the forest at Tasik Bera. *Maxomys rajah* is listed as Vulnerable (IUCN 2016), where the declining population resulted from degradation and habitat loss of lowland forest (Rahim *et al*. 2016). Five individuals of this species were observed in Tasik Bera. This emphasises the importance of protected area as an effective tool in conserving rare species.

*Paradoxurus hermaphroditus* was found at UMT Campus, Tasik Bera and Tasik Kenyir. However, it remains to be documented on Pulau Redang. *P. hermaphroditus* usually inhabits primary forests but occurs at lower densities in secondary and selectively logged forests (Grassman 1998). *P. hermaphroditus* are considered a nuisance in most parts of Malaysia since they litter the ceilings and attics of people’s houses and make loud noises, fighting and moving about at night. This species is a highly frugivorous animal and a legitimate seed disperser (Nakashima *et al*. 2010). Their ability to disperse seeds over long distances is vital for the sustainability of plant populations in degraded forests as well as the recovery of vegetation. The IUCN Red List of Threatened Species classifies *P. hermaphroditus* as a species of Least Concern (LC), indicating that this species receives relatively minimal conservation attention because its population is considered abundant, and it is far from facing the threat of extinction (Duckworth *et al*. 2016).

This study underscores the critical conservation status of *Presbytis femoralis*, classified as Critically Endangered (CR) and *Panthera pardus*, classified as Vulnerable (VU) according to the IUCN Red List of Threatened Species. These species were exclusively recorded at Tasik Bera. *P. femoralis* is typically observed living in groups of three to six members, with a preference for trees of the family Dipterocarpaceae. The combined expert assessment revealed *P. femoralis* optimal distribution pattern across Johor and Pahang, where it inhabits a spectrum of ecosystems ranging from pristine lowland forests and peat swamps to anthropogenically modified landscapes (Haris *et al*. 2024). This revised distribution map gained further validation through targeted interviews and systematic surveys conducted with Orang Asli communities in Tasik Bera, which documented the species’ cultural significance and utilisation in indigenous practices—from traditional cuisine and ceremonial activities to ethnomedicine and artisanal craftsmanship. In contrast, *P. pardus* is a solitary animal that exhibits diurnal activity patterns, often seen even at mid-day in Tasik Bera. This species tends to favour the lower forest canopy as its comfort zone, frequently foraging in this area and descending head-first from the canopy. Additionally, six other species are classified as Endangered (EN) according to the IUCN Red List of Threatened Species (2023), *Nycticebus coucang*, *Tachypithecus obscurus*, *Macaca nemestrina*, *M. fascicularis*, *Elephas maximus* and *Tapirus indicus*. Currently, these species are threatened by deforestation, habitat fragmentation, land conversion, habitat loss, anthropogenic activities, urbanisation and land clearing for agriculture (Dzulhelmi *et al*. 2019; Lim *et al*. 2022; Menon & Tiwari 2019; Najmuddin *et al*. 2019; 2020; Nekaris & Nijman 2007). These factors collectively contribute to the ongoing decline of these species over the years, primarily attributable to habitat loss. *P. femoralis* and *T. obscurus* prefer dipterocarp forests, including lowland and hill forests. These primates are arboreal and depend on the forest canopy for feeding and movement. *P. femoralis* can be found in southern part of Pahang, a small population in Singapore, and fewer than 500 individuals in Johor areas that are herbivores and consume fruits and seeds (Najmuddin *et al*. 2020). *T. obscurus* spends much more time feeding and resting than moving. They fully utilise the natural habitat rather than *M. fascicularis*, which forage human settlements (Ruslin *et al*. 2014).

*Elephas maximus* was only recorded at Tasik Bera, a habitat that offers suitable conditions and an ample food supply for this species. This species requires large home ranges and highly depends on forest habitats for survival. However, due to deforestation, *E. maximus* shifted their diet to eating grasses by the side of the road because of restricted movements, which eventually led to human-wildlife conflicts (Bahar *et al*. 2018; Yamamoto-Ebina *et al*. 2016). In areas where elephants are present, safeguarding against them can be a major reason for constructing tree houses. Thus, Orang Asli Semelai at Tasik Bera sometimes build shelters above the ground, typically as temporary refuges from elephants and as a method of protecting crops. Although Afiq Ramlee (2020) previously documented the presence of *E. maximus* at Tasik Kenyir, no individuals from this species were recorded in the current study. This disparity may be attributed to the sampling being conducted in the peripheral areas of Tasik Kenyir. In contrast, the elephant population typically concentrates near the lake.

*Tapirus indicus* was also exclusively documented at Tasik Bera. *T. indicus* prefers forested environments, especially lowland and swamp forests. Their presence is primarily associated with proximity to water sources, and they necessitate a combination of forested areas and open spaces for activities such as feeding, reproduction and movement (Mohamed & Traeholt 2010; Samantha *et al*. 2020), characteristics that align with the habitat characteristics of Tasik Bera. Additionally, this study documented a roadkill incident involving *Viverra tangalunga*, highlighting the impact of human infrastructure on wildlife in the area. *V. tangalunga* has been observed in logged forests and cultivated areas adjacent to Tasik Bera. Known for its adaptability to human activities, this species readily adjusts to anthropogenic landscapes (Vaughan *et al*. 2011).

The rapid assessment conducted in this study manifested the detrimental effects of forest habitat fragmentation, disturbances and lack of connectivity on large mammal species (Zungu *et al*. 2020; Meza-Joya *et al*. 2020), particularly in urban settings and the geographically isolated Pulau Redang, which showcased the lowest number of species. These factors may have led to the collapse of populations, causing a significant decline in the number of large mammal species on this island. Pulau Redang faces unique challenges regarding habitat fragmentation and isolation in the South China Sea. The island’s geographical separation limits the dispersal of all large mammals to medium-sized species, making it more vulnerable to population collapse (Alzate *et al*. 2019). The restricted gene flow and reduced population size of Pulau Redang may increase the risk of inbreeding and genetic impoverishment, further threatening the survival of these remaining species of mammals. The urban setting of the UMT Campus poses numerous challenges for large mammals. The encroachment of human settlements, infrastructure development and habitat destruction result in fragmented habitats, isolating populations and impeding their movements. The limited availability of suitable habitats in urban areas further exacerbates the situation, causing the localised extinction of large mammal species that cannot adapt to suboptimal conditions and constant disturbances (McKinney 2002).

In contrast, the Tasik Bera area emerges as an important population sink for large, medium and small mammals due to its proximity to surrounding industrial agricultural plantations. Despite the pressures from human activities, the Tasik Bera area provides relatively intact habitats that support viable populations of large mammals. Notably, large mammals such as *Elephas maximus*, *Tapirus indicus*, and top predators such as *Panthera pardus* in the Tasik Bera area contribute to an ecologically functionally complete food web ecosystem. These large herbivores and top predators play crucial roles in maintaining ecosystem balance, controlling populations of the herbivorous animals, and influencing vegetation dynamics and nutrient cycling in the tropical rainforest (Pringle *et al*. 2023). Compared to the UMT urban campus and Pulau Redang, Tasik Bera area demonstrates the importance of preserving and protecting suitable habitats for large mammal species. Conservation efforts should minimise habitat fragmentation, enhance connectivity and promote sustainable land-use practices in urban settings. Similarly, measures should be taken to address the isolation and limited connectivity of Pulau Redang to ensure the survival and genetic diversity of existing medium and small mammal populations. Several measures can be taken such as creating wildlife corridors between fragmented habitats, restoring degraded habitats, planting native vegetation and implementing landscape management practices that promote connectivity across the island. Additionally, effective conservation strategies such as community-based conservation programmes, educational campaigns and ecotourism initiatives that can promote sustainable practices and foster stewardship of the island’s natural resources should be implemented to mitigate the threats large mammals face and ensure the long-term survival of these ecologically significant wildlife species.

## LIMITATION OF THE STUDY

The disparities observed between the findings of this study and previous research can be ascribed to discrepancies in methodologies and the extent of sampling efforts. Some earlier studies utilised alternative methodologies and conducted more extensive sampling efforts, which could have influenced the resulting outcomes. For example, Syakirah *et al*. (2000) employed similar methods to this study but extended their research over a longer period (48 days) and utilised a wider range of bait types, including banana slices, oil palm fruits, dried coconut kernel, jackfruit and fish. Their study also encompassed three distinct locations (Tanjung Kuim, Pos Iskandar and Kampung Jelawat). In contrast, Afiq Ramlee *et al*. (2020) expanded their study by incorporating pitfall traps and conducting research across eight different locations within Tasik Kenyir. Meanwhile, Samsudin (2007) utilised 57 cage traps baited with charred coconut and biscuits with peanut butter, conducting their study at two specific sites within the UMT Campus. The supplementary efforts undertaken in these studies likely enhanced the quality of their outcomes. However, in this study, logistical hurdles were encountered, primarily stemming from limited equipment availability due to constraints in human resources. These limitations impeded the ability to install additional traps across the sampling sites. Moreover, some areas within the sampling site proved particularly challenging to access, thereby restricting overall sampling efforts. Additionally, adverse weather conditions including heavy rainfall, floods and strong winds, impacted both the accuracy of data collection and the safety of the field team. For instance, during the sampling period at Tasik Kenyir, frequent evening-to-night rains were prevalent. Similarly, Tasik Bera experienced three consecutive days of rainfall, leading to flooding. However, trapping endeavours were ultimately successful thereafter. Meanwhile, Pulau Redang and UMT Campus encountered strong windy conditions throughout the sampling periods. Moreover, limited availability of food resources such as fruits may also play a role in contributing to low species diversity (Shukor 2001; Butler & Lawrence 2019). Non-volant mammals including rodents are known for their high mobility, and their distribution patterns are influenced by factors such as altitude, vegetation types and human disturbances (Mulungu *et al*. 2008; Sukma *et al*. 2019).

Despite the findings of this study, several gaps still need to be addressed. One important area that requires further investigation is the assessment of specific habitat requirements for different large mammal species such as *Bos gaurus*, *Panthera pardus* and *Tragulus javanicus*. Understanding the key factors contributing to successful habitat utilisation and movement patterns can guide urban planning and design, ensuring the provision of suitable habitats and wildlife corridors are provided (Zeller *et al*. 2021; Kay *et al*. 2022). Furthermore, research should focus on identifying and quantifying the impacts of anthropogenic disturbances such as noise pollution, light pollution and human-wildlife conflicts on large mammal populations in urban areas (Sordello *et al*. 2020; Morelli *et al*. 2023; Wierucka *et al*. 2023). In geographically isolated areas like Pulau Redang, urgent research is needed to assess the feasibility and effectiveness of reintroduction programmes for large mammal species. This includes investigating potential translocation sites, evaluating the ecological carrying capacity of the island, and assessing the potential impacts of reintroduced species on the island’s ecosystem dynamics. Reintroducing large mammals such as *Tragulus javanicus* and *T. kanchil* into these areas could help restore ecological balance by promoting plant-soil interactions, seed dispersal, nutrient cycling and trophic cascades, and promoting biodiversity (Haynes 2012; Bardgett & Wardle 2003). Additionally, these programmess are crucial for the conservation of endangered or threatened species, aiding in their population recovery and genetic diversity (Weeks *et al*. 2017). Such studies can provide valuable insights into the broader value of wildlife conservation and help garner support for conservation initiatives.

## CONCLUSION

Tasik Bera exhibited the highest diversity of non-volant mammals among the study areas, boasting 18 species across 10 families documented. In contrast, UMT Campus documented six species, while both Pulau Redang and Tasik Kenyir had four species each. When considering data from previous studies, Tasik Kenyir documented the highest diversity, with 87 species, whereas Pulau Redang and UMT Campus showed lower diversity, with only nine and ten species, respectively. Tasik Bera itself boats a respectable count of 47 species across 16 families. Notably, Tasik Bera’s recent observations unveiled 11 new geographically recorded species. Furthermore, three species were recorded in both this study and previous ones. UMT Campus contributed with four unique species and shared one species with past studies. Conversely, neither unique nor shared species were noted Tasik Kenyir and Pulau Redang compared to prior research. Some species displayed restricted distributions such as *Bos gaurus*, exclusively found at Tasik Kenyir, while *Capricornis sumatraensis* and *Rusa unicolor* were also solely observed at Tasik Kenyir.

This study has made significant contributions to the conservation efforts by documenting several species with crucial conservation statuses. *Presbytis femoralis* is identified as Critically Endangered (CR), underscoring the urgent need for conservation actions. Similarly, *Nycticebus coucang*, *Macaca nemestrina*, *M. fascicularis*, *Elephas maximus*, *Tapirus indicus* and *Trachypithecus obscurus* are all classified as Endangered (EN) according to the IUCN Red List of Threatened Species, highlighting their vulnerable status. *Panthera pardus*, *Maxomys rajah*, *M. whiteheadi*, *Lutrogale perspicillata* and *Arctictis binturong* are listed as Vulnerable (VU), indicating the need for heightened conservation efforts to ensure their survival. By shedding light on the conservation statuses of these species, this study provides valuable information that can inform targeted conservation strategies aimed at protecting these vulnerable and endangered populations. This study also offers valuable insights into the distribution patterns and species composition of non-volant small mammals across four distinct study sites in Malaysia. The observed variations in species diversity highlight the importance of habitat heterogeneity and conservation management in shaping the community structure of non-volant mammals. Future research efforts should focus on elucidating the underlying mechanisms driving these patterns and implementing effective conservation strategies to safeguard the biodiversity of these diverse ecosystems.

## Figures and Tables

**Table 1:** Location of four study sites, area type, sampling date and the coordinates.

Study site	Area type	Sampling date	Coordinates	

			Latitude	Longitude
Tasik Bera	Natural lake	27 February–9 March 2023	102° 36′ 28.8″ E	3° 07′ 49.1″ N
Tasik Kenyir	Man-made lake	21 September–24 September 2022	102° 45′ 40.6″ E	5° 08′ 28.7″ N
Pulau Redang	Island	22 August–25 August 2022	103° 01′ 44.6″ E	5° 46′ 02.1″ N
UMT Campus	Urban mangrove	16 August–19 August 2022 28 August–1 September 2022 10 October–13 October 2022	103° 05′ 29.2″ E	5° 24′ 36.5″ N

**Table 2:** The species composition of non-volant mammal across four study sites, alongside their conservation and protection status as assessed by the IUCN Red List of Threatened Species (IUCN 2023), the Red List of Mammals for Peninsular Malaysia (PERHILITAN 2017) and the Wildlife Conservation Act (WCA 2010).

No.	Order	Family	Species name	Common name	Study site				IUCN (2023)	PERHILITAN (2017)	WCA (2010)

					Tasik Bera	Tasik Kenyir	Pulau Redang	UMT Campus			
1	Artiodactyla	Tragulidae	*Tragulus kanchil*	Lesser Mousedeer	4	0	1	0	LC	LC	P
2			*Tragulus napu*	Greater Mousedeer	4	0	0	0	LC	NT	P
3	Carnivora	Felidae	*Panthera pardus*	Black Leopard	1	0	0	0	VU	EN	TP
4			*Prionailurus bengalensis*	Leopard Cat	6	0	0	0	LC	NT	TP
5		Mustelidae	*Lutrogale perspicillata*	Smooth-Coated Otter	0	0	0	7	VU	LC	TP
6		Viverridae	*Viverra tangalunga*	Malayan Civet	1	0	0	0	LC	LC	TP
7			*Arctictis binturong*	Binturong	0	1	0	0	VU	LC	TP
8			*Paguma larvata*	Masked Palm Civet	0	0	0	1	LC	LC	TP
9			*Paradoxurus hermaphroditus*	Common Palm Civet	1	4	0	9	LC	LC	P
10	Perissodactyla	Tapiridae	*Tapirus indicus*	Asian Tapir	2	0	0	0	EN	EN	TP
11	Primates	Cercopithecidae	*Presbytis femoralis*	Raffles’ Banded Langur	1	0	0	0	CR	NT	NP
12			*Macaca fascicularis*	Long-Tailed Macaque	15	0	10	3	EN	LC	P
13			*Macaca nemestrina*	Southern Pig-Tailed Macaque	3	0	0	0	EN	LC	P
14			*Trachypithecus obscurus*	Dusky Langur	10	4	0	0	EN	NT	P
15		Lorisidae	*Nycticebus coucang*	Sunda Slow Loris	2	0	0	0	EN	NT	TP
16	Proboscidea	Elephantidae	*Elephas maximus*	Asian Elephant	1	0	0	0	EN	VU	TP
17	Rodentia	Muridae	*Leopoldamys sabanus*	Long-Tailed Giant Rat	2	0	0	0	LC	LC	NP
18			*Maxomys rajah*	Rajah Spiny Rat	5	0	0	0	VU	LC	NP
19			*Maxomys whiteheadi*	Whitehead’s Spiny Rat	1	0	0	0	VU	LC	NP
20			*Rattus argentiventer*	Ricefield Rat	2	0	0	0	LC	LC	NP
21			*Rattus exulans*	Pacific Rat	2	0	0	0	LC	LC	NP
22			*Rattus rattus*	House Rat	0	0	0	1	LC	LC	NP
23			*Rattus tiomanicus*	Malaysian Wood Rat	1	0	0	0	LC	LC	NP
25		Sciuridae	*Callosciurus notatus*	Plantain Squirrel	1	0	5	0	LC	LC	NP
			*Ratufa bicolor*	Black Giant Squirrel	0	2	0	0	NT	NT	TP
26	Scandentia	Tupaiidae	*Tupaia minor*	Lesser Treeshrew	1	0	0	0	LC	LC	TP
27			*Tupaia glis*	Common Treeshrew	11	0	1	1	LC	LC	TP
	Total number of individuals			73	11	17	22				
	Total number of species			18	4	4	6				
	Total number of families			9	3	4	5				
	Total number of genera			12	4	4	6				

*Notes*: CR = Critically Endangered; EN = Endangered; VU = Vulnerable; NT = Near Threatened; LC = Least Concern, P = Protected Wildlife; TP = Totally Protected Wildlife; NP = Non-protected Wildlife.

**Table 3:** Species diversity and capture rate estimated for each study locality.

	Tasik Bera	Tasik Kenyir	Pulau Redang	UMT Campus
Species	18	4	4	6
Individuals	73	11	17	22
Trapping effort (Trap – days)	160	40	40	130
Capture rate (No. of individual/Trapping effort)	0.46	0.28	0.43	0.17
Shannon Diversity Index (*H′*)	2.65	1.26	1.01	1.42
Simpson Dominance Index (*D*)	0.91	0.76	0.6	0.74
Evenness (*E′*)	0.86	0.91	0.73	0.80

**Table 4:** Taxonomic checklist of non-volant mammal from Tasik Bera, Tasik Kenyir, Pulau Redang and UMT Campus.

No.	Order	Family	Species name	Common name	Study site			

					Tasik Bera (This study, William-Dee *et al*. 2019, Madinah *et al*. 2011; Syakirah *et al*. 2000)	Tasik Kenyir (This study, Mohammad Noor *et al*. 2019; Nor Zalipah *et al*. 2019)	Pulau Redang (This study, Robinson & Kloss 1911; Malaysian Nature Society 1990; Abdullah *et al*. 2019)	UMT Campus (This study, Samsudin 2007; Anuar 2007; Mohd Nasir 2008)
1	Artiodactyla	Bovidae	*Bos gaurus*	Gaur		x		
2			*Capricornis sumatraensis*	Sumatran Serow		x		
3		Cervidae	*Rusa unicolor*	Sambar		x		
4			*Muntiacus muntjak*	Southern Red Muntjac		x		
5		Suidae	*Sus scrofa*	Wild Boar		x		
6		Tragulidae	*Tragulus javanicus*	Javan Mousedeer	x	x	x	
7			*Tragulus kanchil*	Lesser Mousedeer	x	x	x	
8			*Tragulus napu*	Greater Mousedeer	x	x		
9	Dermoptera	Cynocephalidae	*Galeopterus variegatus*	Sunda Flying Lemur	x	x		
10	Eulipotyphla	Erinaceidae	*Echinosorex gymnura*	Moonrat	x	x		
11			*Hylomys suillus*	Short-tailed Gymnure				
12	Insectivora	Soricidae	*Crocidura fuliginosa*	Southeast Asian Shrew			x	
13			*Crocidura malayana*	Malayan Shrew		x	x	
14			*Suncus etruscus*	Pygmy Whited-tooth Shrew		x		
15	Carnivora	Canidae	*Cuon alpinus*	Dhole		x		
16			*Canis familiaris*	Wild Dog		x		
17		Felidae	*Catopuma temminckii*	Asian Golden Cat		x		
18			*Panthera pardus*	Black Leopard	x	x		
19			*Panthera tigris*	Tiger		x		
20			*Pardofelis marmorata*	Marbled Cat		x		
21			*Prionailurus bengalensis*	Leopard Cat	x	x		
22			*Prionailurus planiceps*	Flat-headed Cat	x	x		
23			*Neofelis nebulosa*	Clouded Leopard		x		
24		Hespertidae	*Herpestes urya*	Crab-eating Mongoose		x		
25		Mustelidae	*Amblonyx cinerea*	Asian Small-clawed Otter	x	x		
26			*Lutrogale perspicillata*	Smooth-coated Otter	x	x		x
27			*Lutra sumatrana*	Hairy-nosed Otter		x		
28			*Martes flavigula*	Yellow-throated Marten		x		
29			*Mustela nudipes*	Malay Weasel		x		
30		Prionodontidae	*Prionodon linsang*	Banded Linsang		x		
31		Ursidae	*Helarctos malayanus*	Sun Bear		x		
32		Viverridae	*Arctictis binturong*	Binturong		x		
33			*Arctogalidia trivirgata*	Small-toothed Palm Civet	x	x		
34			*Hemigalus derbyanus*	Banded Civet		x		
35			*Paguma larvata*	Masked Palm Civet		x		x
36			Paradoxurus hermaphroditus	Common Palm Civet	x	x		x
37			*Viverra megaspila*	Large-spotted Civet		x		
38			*Viverra tangalunga*	Malayan Civet	x	x		
39			*Viverra zibetha*	Large Indian Civet		x		
40	Perissodactyla	Rhinocerotidae	*Dicerorhinus sumatrensis*	Sumatran Rhinoceros		x		
41		Tapiridae	*Tapirus indicus*	Asian Tapir	x	x		
42	Pholidota	Manidae	*Manis javanica*	Sunda Pangolin	x	x		
43	Primates	Cercopithecidae	*Presbytis femoralis*	Raffles’ Banded Langur	x	x		
44			*Presbytis siamensis*	White-thighed Surili		x		
45			*Macaca fascicularis*	Long-tailed Macaque	x	x	x	x
45			*Macaca nemestrina*	Southern Pig-tailed Macaque	x	x		
47			*Trachypithecus obscurus*	Dusky Langur	x	x		
48		Hylobatidae	*Hylobates lar*	Malaysian Lar		x		
49			*Symphalangus syndactylus*	Siamang		x		
50		Lorisidae	*Nycticebus coucang*	Sunda Slow Loris	x			
51	Proboscidea	Elephantidae	*Elephas maximus*	Asian Elephant	x	x		
52	Rodentia	Muridae	*Berylmys bowersi*	Bower’s White-toothed Rat		x		
53			*Lenothrix canus*	Gray Tree Rat	x			
54			*Leopoldamys sabanus*	Long-tailed Giant Rat	x	x		
55			Maxomys inas	Malayan Mountain Maxomys		x		
56			*Maxomys rajah*	Brown Spiny Rat	x	x		
57			*Maxomys surifer*	Red Spiny Rat	x	x		
57			*Maxomys whiteheadi*	Whitehead’s Spiny Rat	x	x		
59			*Mus caroli*	Ryukyu Mouse		x		
60			Mus musculus	House Mouse		x		
61			*Niviventer cremoriventer*	Dark-tailed Tree Rat	x	x		
62			*Niviventer fulvescens*	Chestnut White-bellied Rat		x		
63			*Pithecheir parvus*	Malay Peninsula Pithecheir	x	x		
64			*Rattus annandalei*	Annandale’s Rat		x		
65			Rattus argentiventer	Ricefield Rat	x	x		x
66			*Rattus exulans*	Pacific Rat	x	x		x
67			*Rattus rattus*	House Rat		x		x
68			*Rattus tiomanicus*	Malaysian Wood Rat	x	x	x	x
69			*Sundamys muelleri*	Mueller’s Rat	x	x		
70		Sciuridae	*Aeromys tephromelas*	Black Flying Squirrel		x		
71			*Callosciurus caniceps*	Grey-bellied Squirrel	x	x		
72			*Callosciurus nigrovittatus*	Black-striped Squirrel	x	x		
73			*Callosciurus notatus*	Plantain Squirrel	x	x	x	x
74			*Callosciurus prevostii*	Prevost’s Squirrel		x		
75			*Hylopetes platyurus*	Red Giant Flying Squirrel		x		
76			*Hylopetes spadiceus*	Red-cheeked Flying Squirrel		x		
77			*Lariscus insignis*	Three-striped Ground Squirrel	x	x		
78			*Petaurista petaurista*	Red Giant Flying Squirrel	x	x		
79			*Petinomys setosus*	Temminck’s Flying Squirrel		x		
80			*Petinomys vordermanni*	Vordermann’s Flying Squirrel	x	x		
81			*Ratufa affinis*	Pale Giant Squirrel	x	x		
82			*Ratufa bicolor*	Black Giant Squirrel	x	x		
83			*Rhinosciurus laticaudatus*	Shrew-faced Squirrel	x	x		
84			*Sundasciurus hippurus*	Horse-tailed Squirrel		x		
85			*Sundasciurus lowii*	Low’s Squirrel	x	x		
86			*Sundasciurus tenuis*	Slender Squirrel	x	x	x	
87		Spalacidae	*Rhizomys sumatrensis*	Indomalayan Bamboo Rat		x		
88		Hystricidae	*Atherurus macrourus*	Asiatic Brush-tailed Porcupine		x		
89			*Hystrix brachyura*	Malayan Porcupine	x	x		
90			*Trichys fasciculata*	Long-tailed Porcupine		x		
91	Scandentia	Ptilocercidae	*Ptilocercus lowii*	Pen-tailed Treeshrew	x			
92		Tupaiidae	*Tupaia minor*	Lesser Treeshrew	x			
93			*Tupaia glis*	Common Treeshrew	x	x	x	x
	Total number of species				47	87	9	10
	Total number of genera				34	62	7	7
	Total number of families				16	24	6	6

## References

[b1-tlsr_36-1-127] Abdullah F, Kamarulnizam SA, Suwati M, Iand Ibnu S, Rahim ARA, Koh HL, Kamaruzaman MP, Abdullah M, Latiff A (2011). Beetle fauna of Cameron Highlands montane forest. Siri kepelbagaian biologi hutan, pengurusan hutan, persekitaran fizikal dan kepelbagaian biologi.

[b2-tlsr_36-1-127] Abdullah MT, David G, Ariffin MSA (2019). The mesmerizing Pulau Redang: An introduction to its ecology and biodiversity.

[b3-tlsr_36-1-127] Abdullah MT, Rahim ANA, Pesiu E (2017). The enchanting Pulau Perhentian an introduction to its biodiversity.

[b4-tlsr_36-1-127] Abdullah MT (2013). List of 361 species of mammals in Malaysia.

[b5-tlsr_36-1-127] Afiq Ramlee MN, Mohd Fadli H, Azuan R, Fathihi Hakimi R, Pesiu E, Noor Aisyah AR, Nur Izzah Izzati A, Gertrude D, Amirah Azizah Z, Nur Amalina A, Hasrulzaman HB, Muhammad Syamsul AA, Bartholomew CV, Muhamad Aidil Z, Muhamad Safiih L, Abdullah MT (2020). Conspectus of flora, fauna and micro-climate data in Tasik Kenyir from Mac 2015–February 2016. Data in Brief.

[b6-tlsr_36-1-127] Ahmad Juffiry S, Yusof E, Zakaria M (2015). Diversity of fauna species in Ayer Hitam Forest Reserve, Selangor, Malaysia. The Malaysian Forester.

[b7-tlsr_36-1-127] Alzate A, Etienne RS, Bonte D (2019). Experimental island biogeography demonstrates the importance of island size and dispersal for the adaptation to novel habitats. Global Ecology and Biogeography.

[b8-tlsr_36-1-127] Anuar AA (2007). Ectoparasites composition on small mammals at mangrove area of Universiti Malaysia Terengganu. Undergraduate diss.

[b9-tlsr_36-1-127] Azovsky AI (2011). Species-area and species-sampling effort relationships: Disentangling the effects. Ecography.

[b10-tlsr_36-1-127] Badli Sham BH, Mohd Ibrahim NS, Xian G, Noh H, Shukor A, Shafie F, Mohd Daud N, Abdul Razak FA, Rosli R, Razak A, Mohammad F, Kamaruzzaman M, Mohamad S, Dzu K, Shariffudin A, Mohd Sawawi SN, Ahmad A (2019). Herpetofauna of Universiti Malaysia Terengganu campus: Sustaining biodiversity in Campus Green area. Journal of Sustainability Science and Management.

[b11-tlsr_36-1-127] Bahar A, Abu Kasim N, Hambali K (2018). Home range and movement patterns of Asian elephant (*Elephas maximus*) in Gua Musang, Kelantan, Malaysia. Malayan Nature Journal.

[b12-tlsr_36-1-127] Baharudin NS, Tah MMTM, Zulkifli SZ, Ab Ghani NI, Noor HM, Sabar Sabal NH (2023). Species diversity and distribution of non-volant small mammal between restoration, boundary, disturbed and undisturbed area in Cameron Highlands, Malaysia. Tropical Life Sciences Research.

[b13-tlsr_36-1-127] Baqi A, Azhar I, Chen EW, Khan FAA, Lian CJ, Nelson BR, Kumaran JV (2021). The diversity of small mammals in Pulau Perhentian Kecil, Terengganu, Malaysia. Journal of Threatened Taxa.

[b14-tlsr_36-1-127] Bardgett RD, Wardle DA (2003). Herbivore-mediated linkages between aboveground and belowground communities. Ecology.

[b15-tlsr_36-1-127] Biun A, Mohd Buang M (2014). Diversity and abundance of bird communities in Tasek Bera Ramsar site, Pahang, Malaysia. Journal of Wildlife and Parks.

[b16-tlsr_36-1-127] Butler RA, Lawrence WF (2019). New strategies for conserving tropical forests. Trends Ecology Evolution.

[b17-tlsr_36-1-127] Corlett RT (1992). The ecological transformation of Singapore, 1819–1990. Journal of Biogeography.

[b18-tlsr_36-1-127] Crooks KR, Burdett CL, Theobald DM, King SR, Di Marco M, Rondinini C, Boitani L (2017). Quantification of habitat fragmentation reveals extinction risk in terrestrial mammals. Proceedings of the National Academy of Sciences of the United States of America.

[b19-tlsr_36-1-127] Department of Wildlife and National Parks (2016). Protected areas.

[b20-tlsr_36-1-127] Duckworth JW, Timmins RJ, Choudhury A, Chutipong W, Willcox DHA, Mudappa D, Rahman H, Widmann P, Wilting A, Xu W (2016). Paradoxurus hermaphroditus. The IUCN Red List of Threatened Species: e.T41693A45217835.

[b21-tlsr_36-1-127] Dzulhelmi M, Suriyanti S, Manickam S (2019). Population, behaviour and conservation status of long-tailed macaque, *Macaca fascicularis* and southern pig-tailed macaque, *Macaca nemestrina* in Paya Bakau Park, Perak, Malaysia. JAPS: Journal of Animal and Plant Sciences.

[b22-tlsr_36-1-127] Forestry Department of Peninsular Malaysia (2022). Forestry statistics.

[b23-tlsr_36-1-127] Francis CM (2008). A field guide to the mammals of South-East Asia.

[b24-tlsr_36-1-127] Francis CM (2013). A photographic guide to mammals of South-east Asia: Including Thailand, Malaysia, Singapore, Myanmar, Laos, Cambodia, Vietnam, Java, Sumatra, Bali and Borneo.

[b25-tlsr_36-1-127] Francis CM, Barrett P (2008). Guide to the mammals of Southeast Asia.

[b26-tlsr_36-1-127] Gharibreza M, Raj JK, Yusoff I, Othman Z, Wan Muhamad Tahir WZ, Ashraf MA (2013). Sedimentation rates in Bera Lake (Peninsular Malaysia) using 210Pb and 137Cs radioisotopes. Geosciences Journal.

[b27-tlsr_36-1-127] Ginsberg JR, Levin MA (2013). Mammals, biodiversity of. Encyclopaedia of biodiversity-Biodiversity of mammals.

[b28-tlsr_36-1-127] Gotelli NJ, Entsminger GL (2015). EcoSim: Null Models Software for ecology.

[b29-tlsr_36-1-127] Grassman LI (1998). Movements and fruit selection of two *Paradoxurinae* species in a dry evergreen forest in Southern Thailand. Small Carnivore Conservation.

[b30-tlsr_36-1-127] Gumert M, Malaivijitnond S (2012). Marine prey processed with stone tools by Burmese long-tailed macaques. American Journal of Physical Anthropology.

[b31-tlsr_36-1-127] Hadley AS, Frey SJ, Robinson WD, Kress WJ, Betts MG (2014). Tropical forest fragmentation limits pollination of a keystone understory herb. Ecology.

[b32-tlsr_36-1-127] Hammer Ø, Harper DA, Ryan PD (2001). PAST: Paleontological statistics software package for education and data analysis. Palaeontologia Electronica.

[b33-tlsr_36-1-127] Haris H, Othman N, Kaviarasu M, Najmuddin MF, Abdullah-Fauzi NAF, Ramli FF, Sariyati NH, Ilham-Norhakim ML, Md-Zain BM, Abdul-Latiff MAB (2024). Ethnoprimatology reveals new extended distribution of critically endangered banded langur *Presbytis femoralis* (Martin, 1838) in Pahang, Malaysia: Insights from indigenous traditional knowledge and molecular analysis. American Journal of Primatology.

[b34-tlsr_36-1-127] Haynes G (2012). Elephants (and extinct relatives) as earth-movers and ecosystem engineers. Geomorphology.

[b35-tlsr_36-1-127] Henson IE (1994). Environmental impacts of oil palm plantations in Malaysia.

[b36-tlsr_36-1-127] Holzner A, Ruppert N, Swat F, Schmidt M, Weiß BM, Villa G, Mansor A, Shahrul Anuar MS, Engelhardt A, Kühl H, Widdig A (2019). Macaques can contribute to greener practices in oil palm plantations when used as biological pest control. Current Biology.

[b37-tlsr_36-1-127] Howe HF, Schupp EW, Westley LC (1985). Early consequences of seed dispersal for a neotropical tree (*Virola surinamensis*). Ecology.

[b38-tlsr_36-1-127] IUCN (2023). The IUCN Red List of Threatened Species. Version 2024-2.

[b39-tlsr_36-1-127] IUCN (2016). The IUCN Red List of Threatened Species Version 2016-2.

[b40-tlsr_36-1-127] Jayaraj VK, Daud SHM, Azhar MI, Shahrul Anuar MS, Mokhtar SI, Abdullah MT (2013). Diversity and conservation status of mammals in Wang Kelian State Park, Perlis, Malaysia. Check List.

[b41-tlsr_36-1-127] Jayaraj VK, Tahir NFDA, Udin NA, Baharin NFK, Ismail SK, Zakaria SNA (2012). Species diversity of small mammals at Gunung Stong State Park, Kelantan, Malaysia. Journal of Threatened Taxa.

[b42-tlsr_36-1-127] Kay CAM, Rohnke AT, Sander HA, Stankowich T, Fidino M, Murray MH, Lewis JS, Taves I, Lehrer EW, Zellmer AJ, Schell CJ, Magle SB (2022). Barriers to building wildlife-inclusive cities: Insights from the deliberations of urban ecologists, Urban Planners and Landscape Designers. People and Nature.

[b43-tlsr_36-1-127] Khalib NKA, Nur Juliani S, Basri HH, Nelson BR, Abdullah MT (2018). Non-volant small mammal data from fragmented forests in Terengganu State. Data in Brief.

[b44-tlsr_36-1-127] Kingston T, Francis CM, Akbar Z, Kunz TH (2003). Species richness in an insectivorous bat assemblage from Malaysia. Journal of Tropical Ecology.

[b45-tlsr_36-1-127] Kloss CB (1911). On a collection of mammals and other vertebrates from Terengganu Archipelago. Journal of the F.M. S Museum.

[b46-tlsr_36-1-127] Lacher T, Davidson A, Fleming T, Gomez-Ruiz E, McCracken G, Owen-Smith N, Peres C, Wall S (2019). The functional roles of mammals in ecosystems. Journal of Mammalogy.

[b47-tlsr_36-1-127] Lane DJ, Kingston T, Lee BPH (2006). Dramatic decline in bat species richness in Singapore, with implications for Southeast Asia. Biological Conservation.

[b48-tlsr_36-1-127] Langham NPE (1982). The ecology of the Common tree shrew, *Tupaia glis* in peninsular Malaysia. Journal of Zoology.

[b49-tlsr_36-1-127] Lim BK, Pacheco V, Larsen TH (2016). Small non-volant mammals. Core standardized methods for rapid biological field assessment.

[b50-tlsr_36-1-127] Lim BL (1995). Foods habits of *Tupaia glis* with remarks on the evaluation of its economic importance. The Journal Wildlife and Parks.

[b51-tlsr_36-1-127] Lim QL, Yong CSY, Ng WL, Ismail A, Rovie-Ryan JJ, Rosli N, Inoue-Murayama M, Annavi G (2022). Population genetic structure of wild Malayan tapirs (*Tapirus indicus*) in Peninsular Malaysia revealed by nine cross-species microsatellite markers. Global Ecology and Conservation.

[b52-tlsr_36-1-127] Madinah A, Fatimah A, Mariana A, Abdullah M (2011). Ectoparasites of small mammals in four localities of wildlife reserves in Peninsular Malaysia. Southeast Asian Journal of Tropical Medicine and Public Health.

[b53-tlsr_36-1-127] Malaysian Nature Society (1990). Pulau Redang State Park, terrestrial resources, management problems and proposed land use management guidelines. Final report.

[b54-tlsr_36-1-127] Mariana A, Shukor MN, Norhazizi HM, Nurlemsha BI, Ho TM (2010). Movements and home range of a common species of tree–shrew, *Tupaia glis*, surrounding houses of otoacariasis cases in Kuantan, Pahang, Malaysia. Asian Pacific Journal of Tropical Medicine.

[b55-tlsr_36-1-127] McKinney ML (2002). Urbanization, biodiversity, and conservation: The impacts of urbanization on native species are poorly studied, but educating a highly urbanized human population about these impacts can greatly improve species conservation in all ecosystems. BioScience.

[b56-tlsr_36-1-127] Medway L (1983). The wild mammals of Malaya and Singapore.

[b57-tlsr_36-1-127] Menon V, Tiwari S (2019). Population status of Asian elephants *Elephas maximus* and key threats. International Zoo Yearbook.

[b58-tlsr_36-1-127] Meza-Joya FL, Ramos E, Cardona D (2020). Forest fragmentation erodes mammalian species richness and functional diversity in a human-dominated landscape in Colombia. Mastozoología Neotropical.

[b59-tlsr_36-1-127] Mohamed NZ, Traeholt C (2010). A preliminary study of habitat selection by Malayan tapir, *Tapirus indicus*, in Krau Wildlife Reserve, Malaysia. Tapir Conservation News. IUCN/SSC Tapir Specialist Group.

[b60-tlsr_36-1-127] Mohammad Noor NA, Rahim NA, Ahmad NII, Abdullah MT, Abdullah MT, Mohammad A, Nor Zalipah M, Safiih Lola M (2019). Taxonomic composition of non-volant small mammal assemblages in Tasik Kenyir, Hulu Terengganu, Terengganu. Greater Kenyir landscapes.

[b61-tlsr_36-1-127] Mohd Nasir NF (2008). Non volant mammal diversity and composition at mangrove Universiti Malaysia Terengganu. Undergraduate diss.

[b62-tlsr_36-1-127] Morelli F, Tryjankowski P, Ibáñez-Álamo JD, Díaz M, Suhonen J, Møller AP, Prosek J, Moravec D, Bussière R, Mägi M, Kominos T, Galanaki A, Bukas N, Markó G, Pruscini F, Reif J, Benedetti Y (2023). Effects of light and noise pollution on avian communities of European cities are correlated with the species’ diet. Scientific Reports.

[b63-tlsr_36-1-127] Mulungu LS, Makundi RH, Massawe AW, Machang’u RS, Mbije N (2008). Diversity and distribution of rodent and shrew species associated with variations in altitude on Mount Kilimanjaro, Tanzania. Mammalia.

[b64-tlsr_36-1-127] Mun JSC (2014). Ecology of Long-tailed macaques (*Macaca fascicularis*) and its implications for the management of human-macaque interface in Singapore. PhD diss.

[b65-tlsr_36-1-127] Najmuddin MF, Haris H, Norazlimi N, Md-Zain BM, Mohd-Ridwan A, Shahrool-Anuar R, Husna HA, Abdul-Latiff M (2019). Predation of domestic dogs (*Canis lupus familiaris*) on Schlegel’s banded langur (*Presbytis neglectus*) and crested hawk-eagle (*Nisaetus cirrhatus*) on dusky leaf monkey (*Trachypithecus obscurus*) in Malaysia. Journal of Sustainability Science and Management.

[b66-tlsr_36-1-127] Najmuddin MF, Haris H, Norazlimi N, Md-Zain BM, Mohd-Ridwan A, Shahrool-Anuar R, Husna HA, Abdul-Latiff M (2020). Daily activity budget of banded langur (*Presbytis femoralis*) in Malaysia. Journal of Sustainability Science and Management.

[b67-tlsr_36-1-127] Nakashima Y, Inoue E, Inoue-Murayama M, Sukor AJ (2010). High potential of a disturbance-tolerant frugivore, the Common palm civet *Paradoxurus hermaphroditus* (Viverridae), as a seed disperser for large-seeded plants. Mammal Study.

[b68-tlsr_36-1-127] Nekaris KAI, Nijman V (2007). CITES proposal highlights rarity of Asian nocturnal primates (Lorisidae: *Nycticebus*). Folia Primatologica.

[b69-tlsr_36-1-127] Nor Zalipah M, Roslan A, Senawi J, Jayaraj VK, Azhar MI, Abdullah MT, Lim BL, Abdullah MT, Mohammad A, Nor Zalipah M, Safiih Lola M (2019). Checklist of small mammals of Hulu Terengganu, Terengganu. Greater Kenyir landscapes.

[b70-tlsr_36-1-127] Norfahiah M, Azema I, Marina M, Zakaria M (2012). Status and distribution of non-volant small mammals in Universiti Putra Malaysia, Bintulu Sarawak Campus (UPMKB). Pertanika Journal of Tropical Agriculture Science.

[b71-tlsr_36-1-127] Norfaizal GMM, Masrom H, Muhammad Radzali M (2015). Flora diversity of Pulau Tekak Besar, Tasik Kenyir, Hulu Terengganu, Malaysia. International Journal of Current Research in Biosciences and Plant Biology.

[b72-tlsr_36-1-127] Nowak R (1999). Walker’s mammals of the world.

[b73-tlsr_36-1-127] Nur Syakirah B, Marina MT, Mohd Faris RS, Nur Sa’adah M, Tengku Rinalfi Putra TA (2022). Updated assessment of ground-dwelling mammals in Ayer Hitam Forest Reserve, Selangor. Journal of Sustainability Science and Management.

[b74-tlsr_36-1-127] Osman NA, Abdul-Latiff MAB, Yaakop S, Karuppannan KV, Md-Zain BM (2022). Metabarcoding data analysis revealed the plant dietary variation of long-tailed macaque *Macaca fascicularis* (Cercopithecidae, Cercopithecinae) living in disturbed habitats in Peninsular Malaysia. Biodiversity Data Journal.

[b75-tlsr_36-1-127] Pacini N, Harper DM, Dudgeon D (2008). Aquatic, semi-aquatic and riparian vertebrates. Tropical stream ecology.

[b76-tlsr_36-1-127] Parr JWK (2003). Large mammals of Thailand.

[b77-tlsr_36-1-127] Payne J, Francis CM, Phillipps K (1985). A field guide to the mammals of Borneo.

[b78-tlsr_36-1-127] PERHILITAN (2017). Red list of mammals for Peninsular Malaysia Version 2.0.

[b79-tlsr_36-1-127] Pringle RM, Abraham JO, Anderson TM, Coverdale TC, Davies AB, Dutton CL, Gaylard A, Goheen JR, Holdo RM, Hutchinson MC, Kimuyu DM, Long RA, Subalusky AL, Veldhuis MP (2023). Impacts of large herbivores on terrestrial ecosystems. Current Biology.

[b80-tlsr_36-1-127] Rahim NAA, Ahmad NII, Zakaria AA, Pesiu E, Salam MR, Mamat MA, Abdullah MT (2016). Brief survey of non-volant small mammals on Pulau Perhentian Besar, Terengganu, Malaysia. Journal of Sustainability Science and Management Special Issue.

[b81-tlsr_36-1-127] Ramli R, Hashim R (2009). Diversity of small mammals inhabiting disturbed forest: A case study on Kenaboi Forest Reserve, Jelebu, Negeri Sembilan, Malaysia. Malaysian Journal of Science.

[b82-tlsr_36-1-127] Razali A, Syed Ismail SN, Awang S (2018). Land use change in highland area and its impact on river water quality: A review of case studies in Malaysia. Ecology Process.

[b83-tlsr_36-1-127] Robinson HC, Kloss CB (1911). Crocidura malayana. Journal of the Federated Malay States Museum.

[b84-tlsr_36-1-127] Ruppert N, Mansor A, Shahrul Anuar MS (2015). Diversity and biomass of terrestrial small mammals at a primary rainforest reserve (Segari Melintang Forest Reserve, Peninsular Malaysia. Journal of Tropical Life Science.

[b85-tlsr_36-1-127] Ruslin F, Salmah Y, Badrul Munir MZ (2014). A preliminary study on activity budget, daily travel distance and feeding behaviour of Long-tailed macaques and Spectacled dusky leaf monkey in Bangi campus of Universiti Kebangsaan Malaysia, Selangor. The 2014 UKM FST Postgraduate Colloquium.

[b86-tlsr_36-1-127] Saiful AA, Idris AH, Rashid YN, Tamura N, Hayashi F (2001). Home range size of sympatric squirrel species inhabiting a lowland dipterocarp forest in Malaysia. Biotropica.

[b87-tlsr_36-1-127] Samantha LD, Tee SL, Kamarudin N, Lechner AM, Azhar B (2020). Assessing habitat requirements of Asian tapir in forestry landscapes: Implications for conservation. Global Ecology and Conservation.

[b88-tlsr_36-1-127] Samsudin ZH (2007). Small mammal diversity at mangrove area of Universiti Malaysia Terengganu. Undergraduate diss.

[b89-tlsr_36-1-127] Shahfiz MA, Shahrul Anuar MS, Shukor MN, Nor Zalipah M, Pan KA, Mulin MA, Yusof MO, Khairul NAM, Edzham SMSH, Ganesan M, Nordin A, Juliana S, Stephanie C, Saharil AA, Fadhil AR, Nik Fathul R (2011). Survey of small mammals at two forest reserves in Cameron Highlands.

[b90-tlsr_36-1-127] Sharip Z, Zakaria S, Sengupta M, Dalwani R (2008). Lakes and reservoir in Malaysia: Management and research challenges. Proceedings of Taal 2007: The 12th World Lake Conference.

[b91-tlsr_36-1-127] Shukor MN (2001). An elevational transect study of small mammals on Mount Kinabalu, Sabah, Malaysia. Global Ecology and Biogeography.

[b92-tlsr_36-1-127] Sordello R, Ratel O, Lachapelle FFD, Leger C, Dambry A, Vanpeene S (2020). Evidence of the impact of noise pollution on biodiversity: A systematic map. Environmental Evidence.

[b93-tlsr_36-1-127] Struebig MJ, Kingston T, Zubaid A, Mohd-Adnan A, Rossiter SJ (2008). Conservation value of forest fragments to Palaeotropical bats. Biological Conservation.

[b94-tlsr_36-1-127] Sukma H, Stefano J, Swan M, Sitters H (2019). Mammal functional diversity increases with vegetation structural complexity in two forest types. Forest Ecology and Management.

[b95-tlsr_36-1-127] Syakirah S, Zubaid A, Prentice C, Lopez A, Azmin M, Yusof AM (2000). A small-mammal survey at Tasek Bera, Pahang, Malaysia’s first Ramsar site. Malayan Nature Journal.

[b96-tlsr_36-1-127] Tamblyn A, Turner C, O’Mally R, Weaver N, Hughes T, Hardingham S, Roberts H (2005). Malaysia tropical forest conservation project report of the Perhentian phase 2005.

[b97-tlsr_36-1-127] Tingga RCT, Anwarali FA, Mohd Ridwan AR, Senawi J, Abdullah MT (2012). Small mammals from Kuala Atok, Taman Negara Pahang, Malaysia. Sains Malaysiana.

[b98-tlsr_36-1-127] Vander Wall SB (1990). Food hoarding in animals.

[b99-tlsr_36-1-127] Vaughan TA, Ryan JM, Czaplewski NJ (2011). Mammalogy. Journal of Mammalogy.

[b100-tlsr_36-1-127] Weeks AR, Heinze D, Perrin L, Stoklosa J, Hoffmann AA, Van Rooyen A, Kelly T, Mansergh I (2017). Genetic rescue increases fitness and aids rapid recovery of an endangered marsupial population. Nature Communication.

[b101-tlsr_36-1-127] Wells K, Bagchi R (2005). Eat it or take away-seed predation and removal by rats (Muridae) during a fruiting event in a dipterocarp rainforest. The Raffles Bulletin of Zoology.

[b102-tlsr_36-1-127] Wells K, Corlett RT, Lakim MB, Kalko EKV, Pfeiffer M (2009). Seed consumption by small mammals from Borneo. Journal of Tropical Ecology.

[b103-tlsr_36-1-127] Wierucka K, Hatten CER, Murphy D, Allcock JA, Andersson AA, Bojan JWN, Kong TC, Kwok JK, Lam JYK, Ma CH, Phalke S, Tilley HB, Wang RS, Wang Y, Webster SJ, Mumby HS, Dingle C (2023). Human-wildlife interactions in urban Asia. Global Ecology and Conservation.

[b104-tlsr_36-1-127] Wildlife Conservation Act (WCA) (2010). Act 716: Laws of Malaysia.

[b105-tlsr_36-1-127] William-Dee J, Khan FAA, Rosli Q, Morni MA, Azhar I, Lim LS, Tingga RCT, Rahman MRA (2019). Comparative distribution of small mammal diversity in protected and non-protected area of Peninsular Malaysia. Tropical Life Sciences Research.

[b106-tlsr_36-1-127] Witmer G, Sayler R, Huggins D, Capelli J (2007). Ecology and management of rodents in no-till agriculture in Washington, USA. Integrative Zoology.

[b107-tlsr_36-1-127] Yamamoto-Ebina S, Saaban S, Campos-Arceiz A, Takatsuki S (2016). Food habits of Asian elephants *Elephas maximus* in a rainforest of northern peninsular Malaysia. Mammal Study.

[b108-tlsr_36-1-127] Yasuda M, Miura S, Hussein NA (2000). Evidence for food hoarding behaviour in terrestrial rodents in Pasoh Forest Reserve, a Malaysian lowland rain forest. Journal of Tropical Forest Science.

[b109-tlsr_36-1-127] Yletyinen S, Norrdahl K (2008). Habitat use offield voles (*Microtus agrestis*) in wide and narrowbuffer zones. Agriculture, Ecosystems and Environment.

[b110-tlsr_36-1-127] Zahidin MA, Mohd Zakir NFW, Mohamad Nasir NN, Samiran NA, Razali NH, Zulkarnain NKI, Basri HH, Ramlee MNA, Mamat MA, Abdullah MT, Ong MC, Martin MB, Nurulnadia MY, Wahizatul AA (2022). Rapid assessment of terrestrial fauna in Bidong Island, Malaysia. Bidong Island.

[b111-tlsr_36-1-127] Zakaria M, Nordin M (1998). Comparison of visitation rates of frugivorous birds in primary and logged forest in Sabah lowland dipterocarp forest. Tropical Biodiversity.

[b112-tlsr_36-1-127] Zakaria M, Silang S, Mudin R (2001). Species composition of small mammals at the Ayer Hitam Forest Reserve, Puchong, Selangor. Pertanika Journal of Tropical Agricultural Science.

[b113-tlsr_36-1-127] Zeller KA, Wattles DW, Conlee L, Destefano S (2021). Response of female black bears to a high-density road network and identification of long-term road mitigation sites. Animal Conservation.

[b114-tlsr_36-1-127] Zungu MM, Maseko MST, Kalle R, Ramesh T, Colleen T, Downs CT (2020). Effects of landscape context on mammal richness in the urban forest mosaic of EThekwini Municipality, Durban, South Africa. Global Ecology and Conservation.

